# Transient Radiation Imaging Based on a ZnO:Ga Single-Crystal Image Converter

**DOI:** 10.1038/s41598-018-22615-z

**Published:** 2018-03-08

**Authors:** Mengxuan Xu, Liang Chen, Zhiming Yao, Shuqing Ren, Yuying Zhang, Feng Huang, Xu Ji, Chaohui He, Leidang Zhou, Jing Hu, Shiyi He, Kuo Zhao, Xiaoping Ouyang

**Affiliations:** 1 Xi’an Jiaotong University, School of Nuclear Science and Technology, Xi’an, 710049 P. R. China; 2 Northwest Institute of Nuclear Technology, State Key Laboratory of Intense Pulsed Radiation Simulation and Effect, Xi’an, 710024 P. R. China; 3 Sun Yat-sen University, School of Materials, State Key Laboratory of Optoelectronic Materials and Technologies, Guangzhou, 510275 P. R. China; 4 Tsinghua University, Department of Engineering Physics, Beijing, 100084 P. R. China; 5 Xi’an Research Institute of Hi-Tech, Xi’an, 710025 P. R. China

## Abstract

A ZnO:Ga single crystal with an applicable size of φ40 × 1 mm was prepared using the hydro-thermal method. The crystal exhibits good crystallinity and scintillation properties with a 63.94-arcsec full-width at half-maximum (FWHM) in the X-ray rocking curve (XRC) spectrum, 8% luminous non-uniformity, emission at 389 nm in the X-ray excited luminescence spectrum, fast response and 5.5% BGO luminous intensity. Furthermore, an X-ray pinhole imaging system of nanosecond temporal resolution with a ZnO:Ga single-crystal image converter was established to diagnose the cathode electron emission spatial distribution of an intense current diode. Results for shutter times of 4 μs and 5 ns were obtained, which directly represent the cathode electron spatial distribution throughout the entire pulse duration and during a certain moment of the pulse, respectively. The results demonstrate that the large ZnO:Ga single crystal can diagnose the spatial distribution of cathode electron emission in an intense current diode with high temporal resolution and provide new solutions for high-temporal-resolution diagnosis of a pulse radiation field.

## Introduction

X-ray transient radiation imaging^1^, which is a method for intuitively obtaining information on ultra-fast physical processes, has been considered for use in the fields of flash photography^2^, inertial confinement fusion (ICF)^3^ and high-energy-density physics (HEDP)^4^. In these fields, the duration of the X-ray pulse generated by the diagnosed object (diode, Z-pinch) is commonly tens of nanoseconds or less^5,6^. Therefore, it would be highly beneficial to establish an ultra-high-temporal-resolution X-ray diagnostic system. Generally, X-ray pinhole imaging^7^ is commonly used to diagnose a transient X-ray radiation field, and its temporal resolution, which is a key parameter of the imaging system, is directly influenced by the temporal resolution of the image converter. Consequently, a fast-response image converter is an indispensable part of an ultra-high-temporal-resolution X-ray pinhole imaging system.

The commonly used image converters can be divided into two types: inorganic (such as LaBr_3_ and cerium-doped lutetium oxyorthosilicate (LSO)) and organic (such as BC-408). Inorganic image converters such as LaBr_3_ and LSO are not appropriate for ultra-fast physical processes because of their slow response time^8,9^. Organic image converters, such as BC-408, are not suitable for X-ray detection because of their low density and atomic number^10,11^. In contrast, ZnO has been considered a potential material for transient radiation imaging: it has been known as an ultra-fast semiconductor scintillator since the 1960s^12^ and has been used as phosphors in cathode ray tube (CRT) imaging^13^. However, there has been difficulty in obtaining high-quality ZnO single crystals because of their poor growth process, which restrains their application in radiation detection. With the development of the crystal growth processes in the 21st century, many studies have been reported on the theory research and application of ZnO to radiation detection. Ye *et al*.^14^ and Makino *et al*.^15^ reported the influence of Ga doping on optical properties of ZnO. Simpson *et al*.^16^ reported the 40% NaI luminous intensity of ZnO:In; Kano *et al*.^17^ studied the relationship between the luminescence intensity and the time response of different doped ZnO materials; Neal *et al*.^18^ reported the use of ZnO:Ga scintillators as alpha detectors; and Nakazato *et al*.^19,20^ reported the potential use of ZnO as a high-spatial resolution imaging device. Meanwhile, high-quality, large-size ZnO crystals have been successfully obtained: Ohshima *et al*.^21^ acquired 2-inch ZnO single crystals, and Tanaka *et al*.^22^ obtained 3-inch ZnO single crystals using the hydro-thermal method. These improvements render ZnO a promising candidate as an image converter.

In this paper, a ZnO:Ga single crystal with an applicable size of φ40 × 1 mm was prepared. Based on this crystal, an ultra-high-temporal-resolution X-ray pinhole imaging system was constructed. The cathode electron emission spatial distribution of an intense current diode was diagnosed, and favorable results were achieved.

## Material and Scintillation Properties

**Figure 1:**
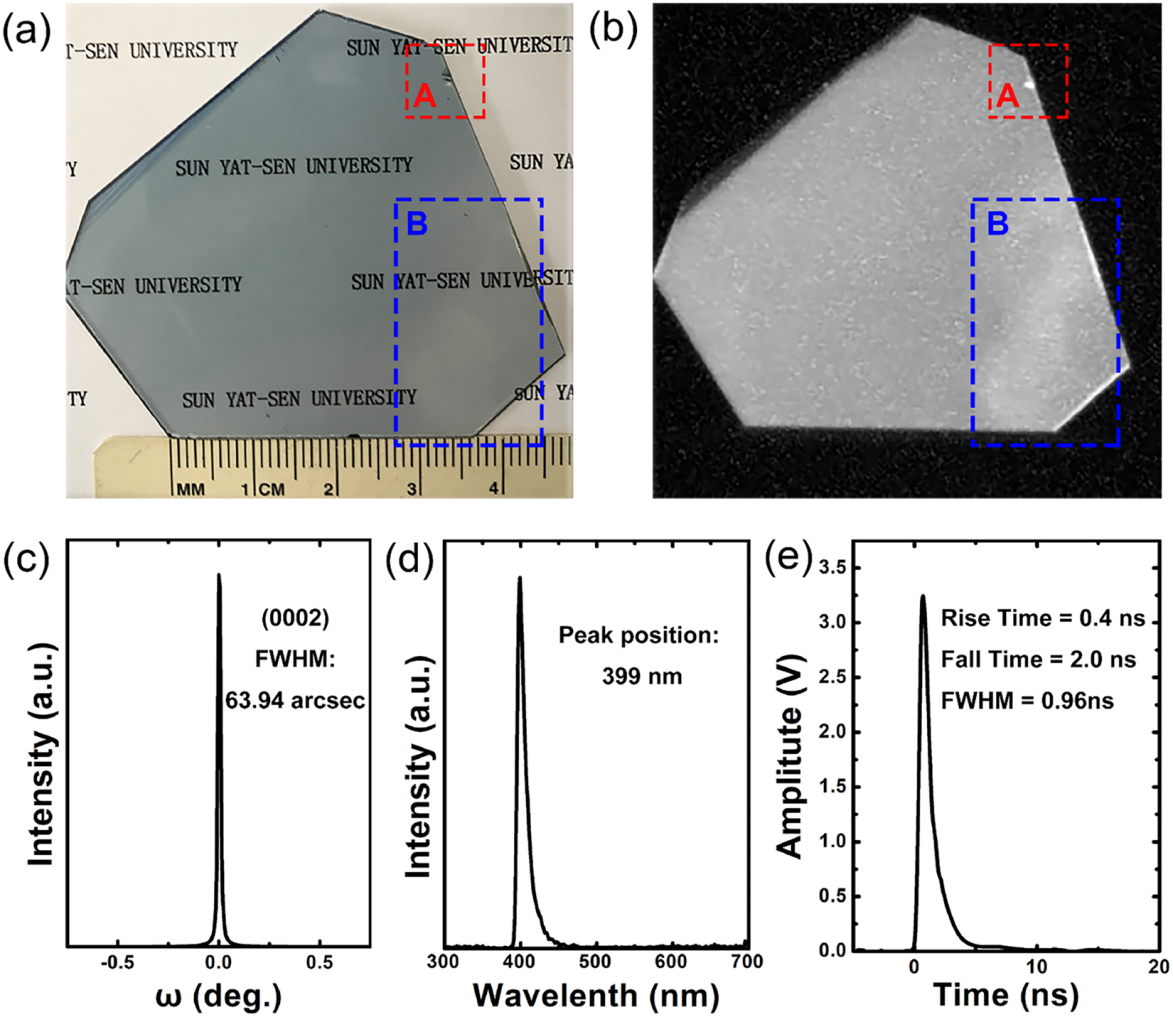
(**a**) Photograph of a ZnO:Ga single crystal grown using the hydro-thermal method; (**b**) luminescence distribution of the ZnO:Ga single crystal excited using X-rays; (**c**) XRC of the ZnO:Ga single crystal; (**d**) X-ray excitation spectrum (XEL) of the ZnO:Ga single crystal at room temperature; (**e**) The time-response of a ZnO:Ga single crystal excited using the pulse γ-ray.

In this work, a ZnO:Ga single crystal was obtained using the hydro-thermal method^23,24^, as shown in Fig. 1(a). The crystal has an applicable size of φ40 × 1 mm and appears pale blue and transparent. Figure 1(b) shows the luminescence distribution excited using DC X-rays. The luminous non-uniformity appears in two major regions (A and B), whereas the remaining region exhibits good uniformity. A comparison between Fig. 1(a,b) shows that the non-uniformity in region A may result from a surface defect caused by machining processes such as cutting and polishing, and the non-uniformity in region B may be related to the crystal growth, based on the lighter color in this zone than that of the surrounding region. We define the luminous non-uniformity N as N = σ/G × 100%, where σ and G are the standard deviation and average value of the surface gray scale, respectively; for this crystal, N = 8%.

Figure 1(c) shows the X-ray rocking curve (XRC) of the ZnO:Ga single crystal. An omega scan was performed for the reflection from the (0002) surface. The full-width at half-maximum (FWHM) was measured to be 63.94 arcsec, indicating the good crystallinity of the ZnO:Ga single crystal. The X-ray excitation spectrum (XEL) of the ZnO:Ga single crystal was measured at room temperature, and the results are shown in Fig. 1(d). The XEL peak of the ZnO:Ga single crystal at room temperature occurs at 399 nm. Figure 1(e) shows the time-response waveforms of the ZnO:Ga single crystal excited using the pulse γ-ray (FWHM of approximately 3 ps^25–27^ and average energy of approximately 10 MeV) which is generated by bombarding iron with the 60 MeV high energy pulse electron beam produced using the electron linear accelerator based on a photocathode RF gun. As shown in Fig. 1(e), the rise time, fall time and FWHM are 0.4 ns, 2.0 ns and 0.96 ns, respectively, indicating the fast response of the crystal. Using a pulsed X-ray source (GOLDEN XRS-4) to excite the BGO^28^ (bismuth germinate, standard reference scintillator, thickness: 1 mm; light yield: 8000 phs/MeV) and ZnO:Ga, and the luminescence was recorded using an intensified charge coupled device (ICCD) camera (Andor, DH34-18F-63). By comparing the grayscale recorded in the ICCD camera, the light output (or luminous intensity) of ZnO:Ga can be measured. The results are shown in Table 1. The net grayscale of BGO and ZnO:Ga are 12996.2 and 713.7, respectively, Therefore, the light output (or luminous intensity) of ZnO:Ga is approximately 5.5% (713.7/12996.2 × 100%) that of BGO.

**Table 1:** The net grayscale of BGO and ZnO:Ga.

Scintillator	Net Grayscale
BGO	12996.2
ZnO:Ga	713.7

### Experimental Procedures

As an intense current electron accelerator, FLASH II^29^ can produce a large-region strong-pulse hard X-ray radiation field^30^, which is of great significance in the study of system-generated electromagnetic pulses (SGEMPs)^31,32^. By diagnosing the produced radiation field, the electron emission on the cathode surface can be obtained, from which the X-ray radiation field uniformity can be evaluated. Therefore, the accelerator operation state can be analyzed, providing a basis for improving the stability of the large-region intense-pulse hard X-ray radiation field. Using the ZnO:Ga single crystal, we constructed a pinhole imaging system, diagnosed the large-region pulsed X-ray radiation field (FWHM of approximately 51 ns, average energy of approximately 86 keV, and average dose of approximately 296 rad(Si)^32^) generated using the FLASH II accelerator, and obtained the spatial distribution of the electron emission at the cathode surface.

**Figure 2:**
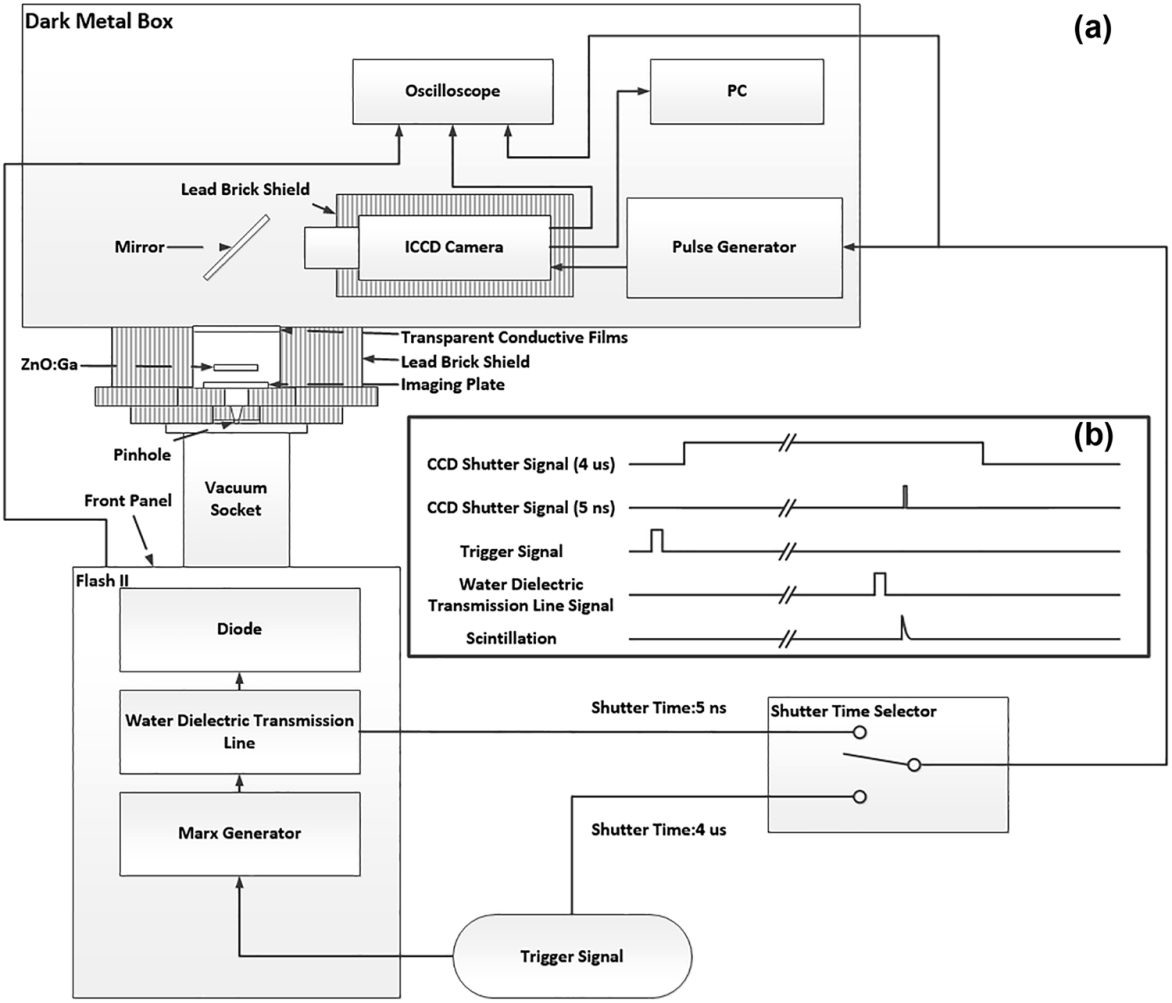
(**a**) Diagnostic system; (**b**) trigger timing of the experiment.

The diagnostic system is shown in Fig. 2(a). The system consists of the FLASH II accelerator, a vacuum socket, a tungsten pinhole (φ = 1 mm), a ZnO:Ga image converter, a mirror, an ICCD camera (Andor, DH34-18F-63), a computer, a pulse generator (Agilent, 81110 A), an imaging plate (FUJI, FCR IR357) and an oscilloscope (Tektronix, DPO4104B). When the system operates, the trigger signal must simultaneously trigger the accelerator and the X-ray pinhole imaging system. Therefore, the trigger signal should be divided into two signals. One signal triggers the accelerator, passes through the Marx generator and water dielectric transmission line, and applies a high voltage to the graphite cathode. Then, the electrons are emitted from the cathode, bombard the 20-µm tantalum anode and produce X-rays. The produced X-ray excites the ZnO:Ga image converter after passing through the vacuum socket and tungsten pinhole. The other signal triggers the pulse generator and ICCD camera to record the luminescence of the ZnO:Ga image converter. The time relationship of the relevant signal is recorded using the oscilloscope. By adjusting the delay time of the pulse generator and the shutter time of the ICCD camera, the spatial distribution of the graphite cathode electron emission can be evaluated for different time periods.

The trigger timing of the experiment is shown in Fig. 2(b). The time interval from the trigger signal to the X-ray signal is approximately 1.8 μs ± 200 ns, where 200 ns is the accelerator jitter caused by the complex structure of the FLASH II accelerator. When the shutter time is set to 5 ns, the ICCD camera may not able to record the luminescence of the ZnO:Ga image converter because the accelerator jitter is much longer than the 5-ns shutter time, whereas the signal jitter of the water dielectric transmission line is several nanoseconds. Therefore, using the signal from the water dielectric transmission line to trigger the pulse generator and ICCD camera, the luminescence of the ZnO:Ga image converter can be successfully recorded. The results show the time-resolved spatial distribution of the graphite cathode electron emission for the given time period. We also obtained the integrated result for a 4-μs shutter time. Because this shutter time is much longer than the system jitter, we can use the trigger signal to trigger the pulse generator and ICCD camera. In this case, the entire luminescence process can be recorded, and the results show the integrated spatial distribution of the graphite cathode electron emission during the pulse duration.

## Results and Discussion

**Figure 3:**
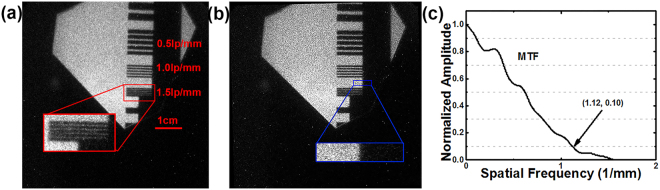
(**a**) The spatial resolution shown by the density strip; (**b**) the knife-edge area; (**c**) the MTF of the system.

Before the experiment, the spatial resolution of the diagnostic system was evaluated. A 10-mm-thick tungsten resolution card was placed in front of the ZnO:Ga image converter. The ZnO:Ga image converter was excited using the pulsed X-ray source (GOLDEN XRS-4), and the luminescence was recorded using the ICCD camera. The spatial resolution of the diagnostic system is shown in Fig. 3(a). A density strip of 1.5 lp/mm can be obtained. The concrete value of the spatial resolution can also be obtained by measuring the modulation transfer function (MTF) of the system^33^.The blue box in Fig. 3(b) shows the knife-edge area, and the MTF obtained is shown in Fig. 3(c), the spatial frequency corresponding to an MTF of 0.1^34^ (MTF10) is equal to 1.12 lp/mm; therefore, the concrete value of the spatial resolution is 1.12 lp/mm, which indicate that the spatial resolution satisfies the requirement of the experiment.

**Figure 4:**
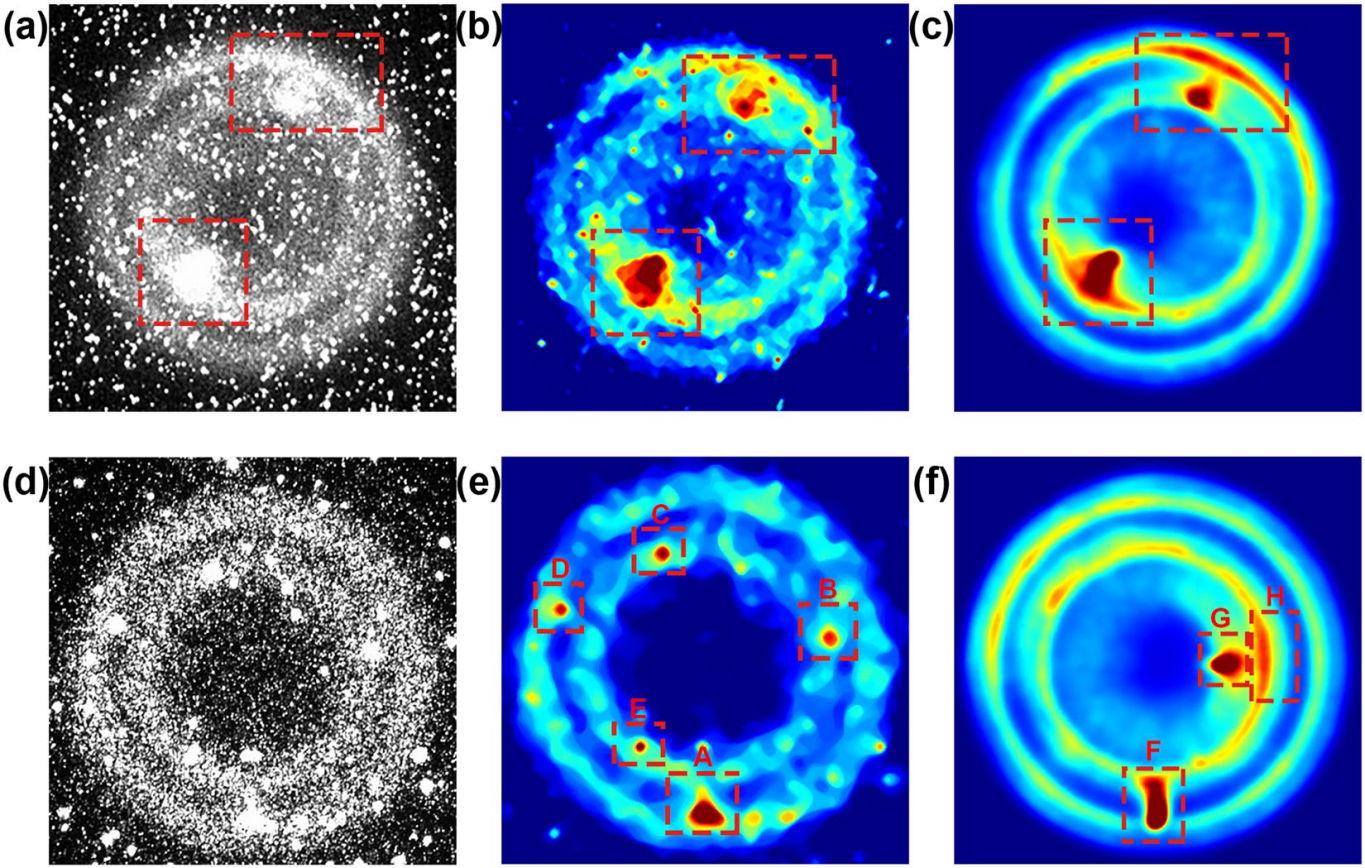
(**a**) Original results of Shot No. 16079 diagnosed using the ZnO:Ga image converter at a 4-μs shutter time; (**b**) filtered results of (**a**); (**c**) time-integrated imaging recorded by the imaging plate (FUJI, FCR IR357) in Shot No. 16079; (**d**) original results of Shot No. 16084 diagnosed by the ZnO:Ga image converter at a 5-ns shutter time; (**e**) filtered results of (**d)**; (**f**) time-integrated imaging recorded by the imaging plate (FUJI, FCR IR357) in Shot No. 16084;

The original results (Shot No. 16079) of the electron emission spatial distribution on the cathode surface for a 4-μs shutter time are shown in Fig. 4(a). The contour of the electron emission region is bicyclic, which is consistent with the shape of the cathode^32^; several pepper-and-salt-like noise spots are also present. Figure 4(b) shows the filtered results of Fig. 4(a). Most of the noise is filtered; some noise remains but it does not affect the result analysis. Figure 4(c) shows the time-integrated imaging results obtained using the imaging plate in Shot No. 16079, which is the same shot shown in Fig. 4(a,b). The results in Fig. 4(b,c) are basically the same, because both of them had recorded the time integrated results in the same shot. The results illustrate that the ICCD camera recorded the time-integrated luminescence process of the ZnO:Ga image converter when the shutter time was set as 4 μs; these results demonstrate the uniformity of the cathode electron emission during the pulse. There are two intense spots in the results, indicating the extremely high intensity and non-uniform distribution of electron emission at the cathode, which negatively affect the X-ray distribution.

Figure 4(d) shows the original results of Shot No. 16084, which were obtained using the ZnO:Ga image converter with a shutter time of 5 ns. The temporal relationship among the water dielectric transmission line signal, the ICCD camera shutter signal and the front-panel signal were measured, and the signal delay caused by the cable was removed. Among these signals, the front-panel signal reflects the time point at which the X-ray arrives at the front panel. We define the moment at which the water dielectric transmission line signal reaches the threshold and triggers the pulse generator as 0 ns. With this reference, the ICCD camera began recording at 120 ns, and the front-panel signal began rising at 101.2 ns. Considering the flight time of the X-rays and the luminescence of the image converter, the time period recorded using the ICCD camera was 13.85–18.85 ns, which is on the rising edge of the X-ray pulse^32^. As a result, transient information regarding the cathode plasma on the rising edge of the pulse can be obtained, but this information cannot be obtained in the integral experiment.

Figure 4(a,d) show that high-gray-scale pepper-and-salt-like noise is always present regardless of the shutter time. The noise arises from two primary causes: the low light yield of ZnO:Ga (therefore the MCP gain of ICCD camera need to be enhanced and that causes more noise, the shutter time and MCP Gain are listed in Table 2) and the excessively high intensity of the X-ray pulse (some X-rays continued scattering into the ICCD camera despite radiation shielding)^35^. When the shutter time was set to 4 μs, the ICCD camera recorded all X-rays scattered during the pulse (about 100 ns^32^), which led to more high-gray-scale pepper-and-salt noise. Comparatively, when the shutter time was set to 5 ns, the amount of pepper-and-salt-like noise decreased because of the shorter shutter time, which allowed for less X-ray scattering and luminescence into the ICCD camera. Therefore, a higher gain is necessary to enhance the signal but results in more noise with a relatively lower grayscale superimposed on the results. As a consequence, the image quality can be improved by strengthening the radiation shielding to reduce the number of scattered X-rays and by enhancing the X-ray sensitivity of the ZnO:Ga image converter to increase the luminous intensity.

**Table 2:** The shutter time and MCP Gain in Shot No. 16079 and 16084.

Shot No.	Shutter Time	MCP Gain
16079	4 μs	150
16084	5 ns	255

Figure 4(e) presents the filtered result of Fig. 4(d). The cathode plasma electron emission surface was formed during 13.85–18.85 ns in Shot No. 16084, and a non-uniform electron emission region appeared but is not obvious. In contrast to Fig. 4(b), a large region of extremely high-intensity electron emission did not appear. Figure 4(f) shows the time-integrated imaging results obtained using the imaging plate in Shot No. 16084, which is the same shot as Fig. 4(d,e). There are three obvious non-uniform regions (F, G and H) in Fig. 4(f). Comparing Fig. 4(f) with Fig. 4(c), we observe that the intense spot position and emission intensity in different shots vary under identical experimental conditions.

Regions A and B in Fig. 4(e) correspond to the same cathode positions as regions F and H in Fig. 4(f), but the emission zones are different, which indicates that the non-uniform emission varies over time. Region G in Fig. 4(f) was not recorded in Fig. 4(e) and shows a new electron emission zone outside the shutter time. Regions C, D and E in Fig. 4(e) are not shown in Fig. 4(f), illustrating that a non-uniform emission spot cannot always further develop into a non-uniform region, which can significantly affect the overall electron emission uniformity. Therefore, non-uniform electron emission can be assumed to be a process, which may occur at any moment in each single pulse duration. In summary, the non-uniform distribution of overall electron emission during the pulse period does not indicate that the electron emission is non-uniform at any moment, and vice versa. Although the distribution of the cathode electron emission at a certain moment was obtained, the appearance and disappearance of non-uniform electron emission occur as a series of changes over time. Thus, it is not sufficient to perform an in-depth study of such a changing process based solely on the result of a single time point. Hence, an ultra-high-temporal-resolution split imaging system is required for in-depth study.

It is generally believed that the process of plasma formation and the state of electron emission from the cathode is highly related to the surface micro-topography of the cathode^36^. When applying the voltage, the electric field at impurities, microprotrusions and sharp edges is much larger than the externally applied electric field, which causes electron emission^37^. In addition, the desorption, vaporization and ionization of surface contaminants at high voltage^38,39^ will also affect the distribution of the electric field and the uniformity of the electron emission distribution. This leads to the degradation of the uniformity of the electron emission distribution. Therefore, one must consider the surface micro-topography and contaminants on the cathode to achieve good uniformity.

As mentioned above, the transient X-ray field produced using an accelerator is always uneven; therefore, a fast diagnostic method is required. Compared to the long-shutter-time integral result, the short-shutter-time differential result reflects the characteristics of the diagnostic object at a specified time of the pulse, which is much more meaningful in terms of diagnosis. We performed a differential diagnosis of pulsed X-rays generated using Flash II and obtained the spatial distribution of the electron emission on the graphite cathode surface at 13.85–18.85 ns. The result directly reflects the uniformity of the cathode electron emission during that time. In comparison, the traditional image converter is restricted by its slow response, and its long afterglow interferes with the diagnostic results. Such interference becomes particularly obvious for a relatively short shutter time and long response time. Because of the fast response of ZnO:Ga in this work, the interference of the afterglow is significantly reduced. Therefore, relative to a traditional image converter, the state of the diagnostic object during the short shutter time is reflected with higher accuracy.

In our work, a ZnO:Ga single crystal was first used as an image converter to diagnose a pulse X-ray radiation field and preliminary results were obtained. The potential for improving the crystal size, scintillation properties and diagnostic system remains. We expect to obtain more accurate and valuable diagnostic results using a larger, faster and higher-radiation-sensitivity crystal, improving the electromagnetic and radiation shielding of the diagnostic system, optimizing the trigger mode and using a split camera.

## Conclusion

In this paper, we applied a single crystal with dimensions of φ40 mm × 1 mm was grown as an image converter to construct an ultra-high-temporal-resolution pinhole imaging system to diagnose the electron emission at the graphite cathode of the diode in FLASH II accelerator. Results were obtained for shutter times of 4 μs and 5 ns with good spatial resolution. The diode cathode electron emission was not absolutely uniform throughout the entire duration or at a given time point in the shot, which is related to the surface micro-topography and contaminants on the cathode. Our results confirm that a ZnO:Ga single crystal can satisfy the requirements of an image converter. More detailed information can be acquired by enlarging the image converter size, enhancing the radiation sensitivity, and constructing a split image system.
